# Heterogeneous Nuclear Ribonucleoproteins A1 and A2 Function in Telomerase-Dependent Maintenance of Telomeres

**DOI:** 10.3390/cancers11030334

**Published:** 2019-03-08

**Authors:** Tong-Hong Wang, Chin-Chuan Chen, Yuan-Chao Hsiao, Yu-Han Lin, Wen-Chieh Pi, Pei-Rong Huang, Tzu-Chien V. Wang, Chi-Yuan Chen

**Affiliations:** 1 Graduate Institute of Health Industry Technology and Research Center for Food and Cosmetic Safety, Research Center for Chinese Herbal Medicine, College of Human Ecology, Chang Gung University of Science and Technology, Tao-Yuan 333, Taiwan; cellww@gmail.com (T.-H.W.); evalin012880@gmail.com (Y.-H.L.); 2 Tissue Bank, Chang Gung Memorial Hospital, Linkou, Tao-Yuan 333, Taiwan; chinchuan@mail.cgu.edu.tw; 3 Graduate Institute of Natural Products, Chang Gung University, Tao-Yuan 333, Taiwan; 4 Department of Molecular and Cellular Biology, College of Medicine, Chang Gung University, Tao-Yuan 333, Taiwan; c11kkh@gmail.com (Y.-C.H.); gratuit777@gmail.com (W.-C.P.); telomerase0701@gmail.com (P.-R.H.); tcvwg@mail.cgu.edu.tw (T.-C.V.W.)

**Keywords:** telomeres, telomerase, hnRNP A1/A2, DNA damage responses, apoptosis

## Abstract

The A/B subfamily of heterogeneous nuclear ribonucleoproteins (hnRNPs A/B), which includes hnRNP A1, A2/B1, and A3, plays an important role in cell proliferation. The simultaneous suppression of hnRNP A1/A2, but not the suppression of hnRNP A1 or A2 alone, has been shown to inhibit cell proliferation and induce apoptosis in cancer cells, but not in mortal normal cells. However, the molecular basis for such a differential inhibition of cell proliferation remains unknown. Here, we show that the simultaneous suppression of hnRNP A1 and hnRNP A2 resulted in dysfunctional telomeres and induced DNA damage responses in cancer cells. The inhibition of apoptosis did not alleviate the inhibition of cell proliferation nor the formation of dysfunctional telomeres in cancer cells depleted of hnRNP A1/A2. Moreover, while proliferation of mortal normal fibroblasts was not sensitive to the depletion of hnRNP A1/A2, the ectopic expression of hTERT in normal fibroblasts rendered these cells sensitive to proliferation inhibition, which was associated with the production of dysfunctional telomeres. Our study demonstrates that hnRNP A1 and A2 function to maintain telomeres in telomerase-expressing cells only, suggesting that the maintenance of functional telomeres in telomerase-expressing cancer cells employs factors that differ from those used in the telomerase-negative normal cells.

## 1. Introduction

Telomeres are specialized structures found at the ends of chromosomes in eukaryotic cells. Human telomeres contain repeats of the sequence CCCTAA/TTAGGG, varying in length from 2 to 50 kilobase pairs, and a short single-stranded G-rich 3′ overhang. DNA is tightly associated with a complex of 6 telomere-specific proteins (POT1, TRF1, TRF2, TPP1, RAP1, and TIN2) known as the shelterin complex, and with surrounding chromatin regulatory factors [1,2,3]. Functional telomeres also play an important role in genome stability [3,4,5], while dysfunctional telomeres activate damage responses mediated by ATM (ataxia telangiectasia, mutated) and ATR (ATM and Rad3-related) kinases, triggering growth arrest, senescence, and apoptosis, as well as increasing end-to-end fusions and anaphase bridges [3]. Dysfunctional telomeres are produced through various mechanisms. For example, telomere attrition represents a normal mechanism that limits the replicative potential of human somatic cells and can serve as a tumor-suppressor pathway in potential cancer cells. Telomere attrition occurs when the ends of linear chromosomes are not completely replicated by normal DNA polymerase. The gradual loss of telomeric DNA with each round of DNA replication subsequently depletes the telomere reserves, resulting in growth arrest accompanied by senescence or apoptosis, a process known as replicative senescence [4,5]. Telomere dysfunction can also arise due to loss or malfunction of telomere-regulating factors. Meanwhile, inhibition or deletion of individual shelterin complex components has been shown to lead to chromosomal abnormalities, cell cycle arrest, and activation of the DNA damage response (DDR) pathway [6,7,8,9].

Heterogeneous nuclear ribonucleoproteins (hnRNPs) constitute a large family of proteins associated with nascent pre-mRNAs, which are packaged into hnRNP particles [10,11,12]. Members of the A/B subfamily of hnRNPs (hnRNP A/Bs), which include hnRNP A1, A2/B1, and A3, are the most abundant hnRNPs in the nucleus of proliferating cells. The major nuclear function of hnRNP A/Bs is thought to involve interactions with RNA, which modulates mRNA packaging, splicing, trafficking, and stability [13,14]. In addition, a role for hnRNP A/Bs in telomere maintenance has also been suggested, since these proteins are able to bind to the single-stranded telomeric repeat sequence of DNA in vitro and have been reported to associate with telomeres in vivo [15,16,17]. It has been demonstrated that hnRNP A1 functions as a positive regulator of telomere length in vivo [15,18], although conflicting results have also been reported [19]. In contrast, hnRNP A3 has been reported as a negative regulator of telomere length maintenance [20]. However, despite these findings, little is known about the functional overlap or antagonism among members of the hnRNP A/B family in telomere maintenance. 

Evidence suggests that hnRNPs A/B play an important role in cell proliferation. For example, the proliferation-dependent expression of hnRNP A1 and A2 has been reported in various cell types [21,22], and while transient RNAi-induced suppression of hnRNP A1, A2, and A3 alone does not appear to affect proliferation, simultaneous suppression of hnRNP A1/A2, A1/A3, or A2/A3 has been shown to significantly reduce the rate of proliferation in a number of different cancer cells [23,24]. Interestingly, the simultaneous suppression of hnRNP A1/A2 has also been shown to induce apoptosis in cancer cells, but not in normal mortal cells [24]. However, the molecular basis of this differential induction of apoptosis in cancer cells remains unknown.

hnRNP A1 and A2 are known to bind to single-stranded telomeric repeat sequences of DNA and to telomeric repeat-containing RNA (TERRA) [16,18,25,26,27]. Such binding activity also affects the binding of other proteins to telomeric G-rich single strands present in the displaced strand of the t-loop structure or in the 3′ overhang [28,29], thereby affecting telomere maintenance. HnRNP A1 has also been shown to act in association with TERRA and POT1 (protection of telomeres 1) to displace RPA (replication protein A) from telomeric single-stranded DNA, suggesting that hnRNP A1 promotes telomere-capping to preserve genomic integrity [30]. Moreover, cells lacking hnRNP A1 exhibit impaired RPA-to-POT1 switching, resulting in DDR at telomeres during mitosis as well as telomere fragility [31]. Although hnRNP A2 is thought to possess activity similar to that of hnRNP A1 in displacing RPA from telomeric single-stranded DNA [30], it is unknown whether they share functional redundancy in telomere maintenance in vivo. In view of the fact that simultaneous suppression of hnRNP A1/A2 is required to inhibit cell proliferation and induce apoptosis in cancer cells, but not in normal mortal cells [24], and the fact that telomerase is expressed in cancer cells but not normal mortal cells, we hypothesized that the hnRNP A1/A2 are involved in telomere maintenance in telomerase-expressing cells. In this study, we employed transient RNA interference (RNAi)-mediated suppression of hnRNP A1 or A2 to determine the role of these two proteins in telomere maintenance and to whether telomere maintenance activity requires the expression of telomerase.

## 2. Results

### 2.1. Induction of DDR by Depletion of both hnRNP A1 and A2 in Cancer Cells

**Figure 1:**
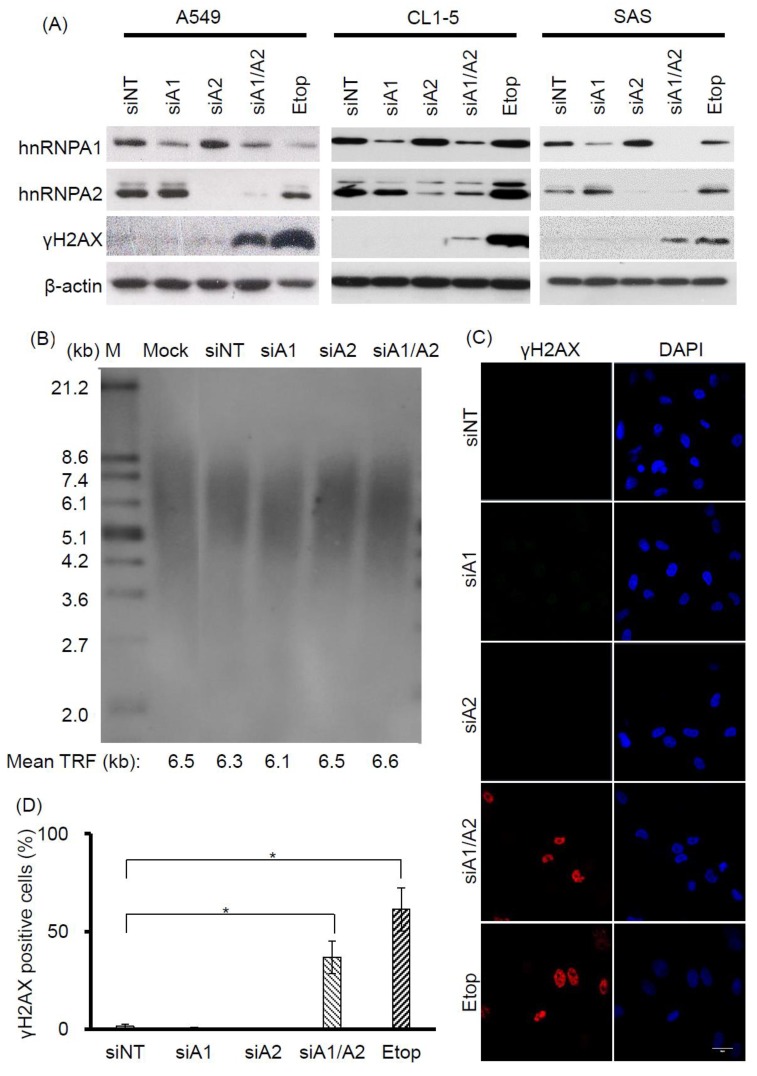
Effects of depleting hnRNP A1 and/or hnRNP A2 on induction of DNA damage response (DDR). (**A**) Induction of γH2AX. A549, CL1-5, and SAS cells were transfected with siRNA targeting hnRNP A1 (siA1), hnRNP A2 (siA2), both hnRNP A1 and A2 (siA1/A2), or with a non-targeting sequence (siNT) for 72 h. Cell lysates were analyzed for expression levels of hnRNP A1, hnRNP A2, and γH2AX by western blotting. β-Actin served as a loading control, and cells treated with 50 μM etoposide (Etop) for 12 h served as a positive control; (**B**) Effect of depleting hnRNP A1 and hnRNP A2 on telomere length in A549 cells. A549 cells were transfected with siRNA targeting hnRNP A1 (siA1), hnRNP A2 (siA2), both hnRNP A1 and A2 (siA1/A2), or with a non-targeting sequence (siNT) for 96 h. The genomic DNA was purified and subjected to telomeric restriction fragment (TRF) length assay, as described in the materials and methods section. Mean TRF length is indicated at the bottom of each lane. Lane M: molecular weight markers. Mock was treated with transfection reagent only; (**C**) A549 cells were fixed and immunostained for γH2AX (red), and nuclei were counterstained with 4′,6-diamidino-2-phenylindole (DAPI, blue). Cells treated with 50 μM etoposide (Etop) for 12 h served as a positive control. The scale bar equals 20 μm. (**D**) The percentage of nuclei showing positive staining for γH2AX was determined from an analysis of ~100 nuclei from each experiment. Positive γH2AX staining is operationally defined here as the detection of 3 or more γH2AX foci in a nucleus. Data shown are mean ± SD from 3 independent experiments. * *p* < 0.05 versus siNT control.

Evidence suggests that hnRNP A1 and A2 share redundant functions in cell proliferation. For example, although transient RNAi-mediated suppression of hnRNP A1 or A2 alone does not appear to inhibit cell proliferation, simultaneous suppression has been shown to reduce the proliferation rate and induce apoptosis in a number of different types of cancer cells [23,24,32]. Moreover, in view of recent findings establishing an important role for hnRNP A1 and A2 in telomere maintenance [30,31], it is likely that the functional redundancy of hnRNP A1 and A2 in cell proliferation may be related to their role in maintaining functional telomeres. To address this, we examined whether DDR is induced by the depletion of hnRNP A1 or A2, since it is known to be triggered by dysfunctional telomeres. Cancer cell lines A549, CL1-5, and SAS were transfected with siRNAs targeting hnRNP A1 and A2, and the expression levels of hnRNP A1, A2, and γH2AX (a DDR marker), were assessed by western blotting. As shown in Figure 1A, treatment of cells with siRNAs targeting hnRNP A1 and hnRNP A2 effectively reduced levels of the targeted proteins. Meanwhile, depletion of hnRNP A1 or A2 alone did not induce γH2AX, unlike the simultaneous suppression of hnRNP A1 and A2 (hnRNP A1/A2), which did so in all 3 cancer cell lines. Under the same experimental conditions, we also confirmed earlier observations that the simultaneous depletion of hnRNP A1 and A2, but not the depletion of hnRNP A1 or A2 alone, is required for the inhibition of cell proliferation and induction of apoptosis in cancer cells [32]. We also revealed that telomere lengths in the hnRNP A1/A2-depleted cells were unaltered (Figure 1B).

To further understand the induction of DDR in hnRNP A1/A2-depleted cells, we chose to conduct detailed studies on one cancer cell line, A549. First, we performed immunofluorescence staining to examine the distribution of γH2AX in A549 cells. Representative results are shown in Figure 1C. γH2AX foci were detected in less than 2% of nuclei of A549 cells transfected with siNT, siA1, or siA2, but were present in about 40% of cells transfected with siA1/A2 (Figure 1D). 

### 2.2. Co-Localization of γH2AX with MDC1 and Telomeres

**Figure 2:**
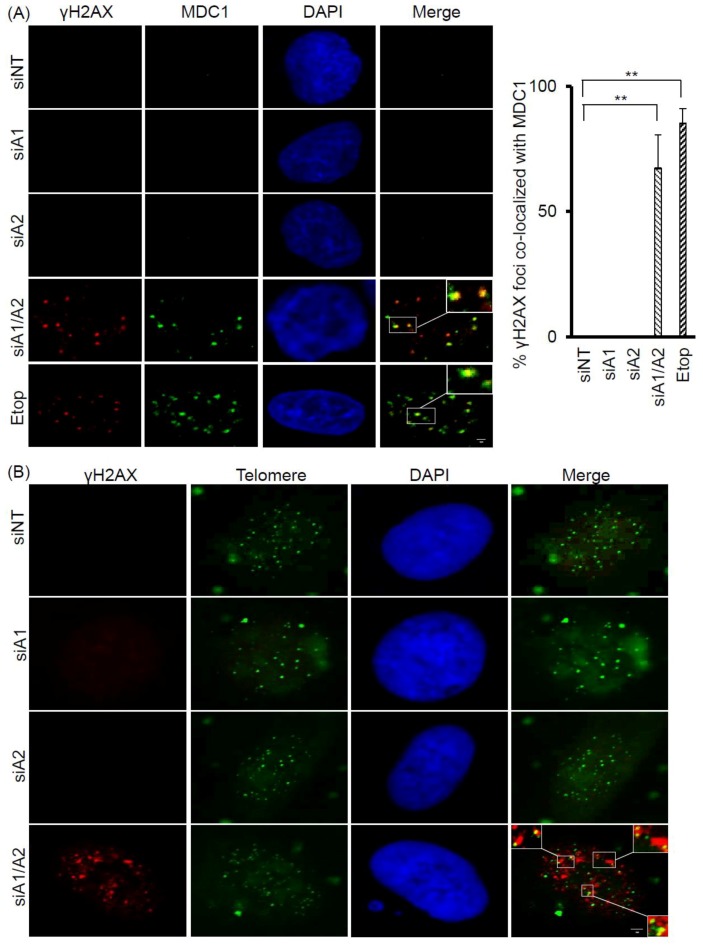
Co-localization of γH2AX with MDC1 and telomere DNA in A549 cells depleted of hnRNP A1/A2. (**A**) A549 cells were transfected with siRNA targeting hnRNP A1 (siA1), hnRNP A2 (siA2), both hnRNP A1 and A2 (siA1/A2), or with a non-targeting sequence (siNT) for 72 h. Cells were fixed and immunostained for γH2AX (red) and MDC1 (green), and nuclei were counterstained with DAPI (blue). A549 cells treated with 50 μM etoposide (Etop) for 12 h served as a positive control. The percentage of γH2AX foci that co-localized with MDC1 (see marked squares for examples) was determined from 50 γH2AX-positive nuclei; data from 3 experiments are summarized in the right panel. ** *p* < 0.01 versus siNT control; The bar equals 2 μm.(**B**) Cells were stained for γH2AX (red) and then for telomeric DNA using FISH with fluorescein isothiocyanate (FITC) -conjugated oligonucleotides (green). γH2AX foci that colocalized with telomere DNA are illustrated in the boxed regions. The scale bar equals 2 μm.

Because γH2AX foci are known to associate with DNA double-strand breaks (DSBs) and DNA DSB repair proteins, we examined whether the γH2AX foci induced in cells depleted of hnRNP A1/A2 also co-localized with MDC1 (mediator of DNA damage checkpoint 1), a DNA DSB repair protein. As shown in Figure 2A, while a weak staining of MDC1 was detected in the nuclei of A549 cells transfected with siNT, siA1, or siA2, strong staining was observed in cells transfected with siA1/A2 and in those treated with etoposide, used as a positive control. More than 60% of γH2AX foci were co-localized with MDC1 foci in cells depleted of hnRNP A1/A2 and in etoposide-treated cells, suggesting that γH2AX foci are associated with DNA DSBs (Figure 2A). 

To test the possibility that dysfunctional telomeres are produced in cells depleted of hnRNP A1/A2, we also examined whether the induced γH2AX foci were co-localized with telomeres. Representative results of the co-localization of γH2AX foci with telomeric DNA are presented in Figure 2B. In A549 cells depleted of both hnRNP A1 and A2, 1–5 γH2AX foci co-localized with telomere DNA in about 40% of γH2AX-positive nuclei.

### 2.3. Failure of Apoptosis Inhibition to Prevent Formation of Dysfunctional Telomeres in Cells Depleted of hnRNP A1/A2

**Figure 3:**
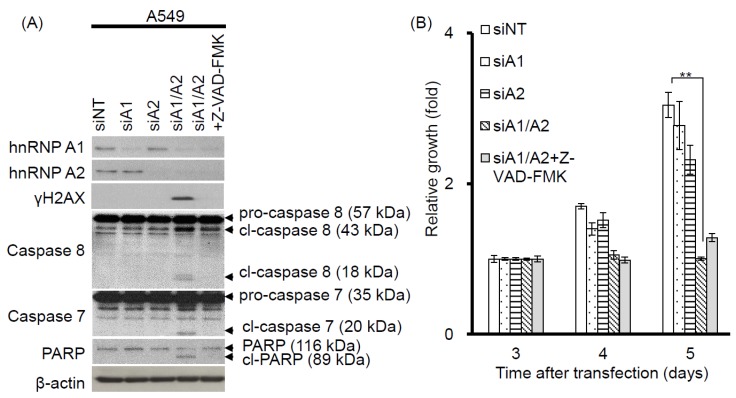

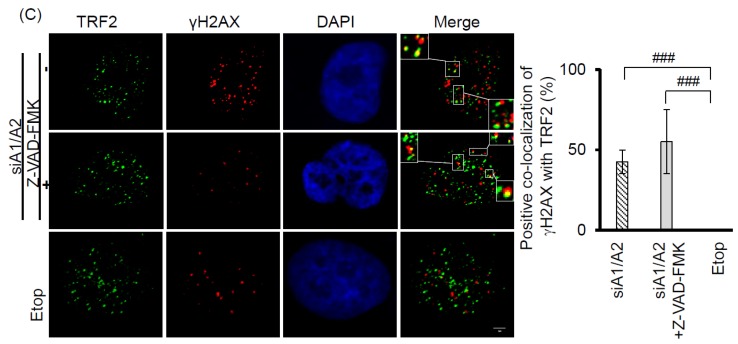
Effects of the apoptosis inhibitor Z-VAD-FMK on the induction of apoptosis, cell proliferation, and DDR. (**A**) A549 cells were transfected with siRNAs targeting hnRNP A1 (siA1), hnRNP A2 (siA2), both hnRNP A1 and A2 (siA1/A2), or with a non-targeting sequence (siNT) for 48 h. Cells transfected with siA1/A2 were cultured in the presence or absence of 20 mM Z-VAD-FMK for 24 h, while other transfected cells were cultured in the absence of Z-VAD-FMK for 24 h. Cell lysates were then analyzed for expression of hnRNP A1, A2, and γH2AX, as well as for the cleavage products of caspase-7 (cl-caspase-7), caspase-8 (cl-caspase-8), and PARP (cl-PARP) by western blotting. β-Actin served as a loading control; (**B**) Treated cells were cultured in regular medium and monitored for cell proliferation using trypan blue staining. The results shown are pooled from 2 independent experiments. ** *p* < 0.01, significant difference from siNT control; (**C**) Cells were fixed and immunostained for γH2AX (red) and TRF2 (Telomeric repeat-binding factor 2, green), and nuclei were counterstained with DAPI (blue). γH2AX foci that co-localized with TRF2 are indicated in the boxed regions. A549 cells treated with 50 μM etoposide (Etop) for 12 h served as a positive control. The percentage of nuclei showing co-localization of γH2AX with TRF2 was determined from analysis of 50 γH2AX -positive nuclei from each experiment. The results shown in the right panel are mean ± SD from 3 independent experiments. ### *p* < 0.001, significant difference from etoposide control. The scale bar equals 2 μm.

**Figure 4:**
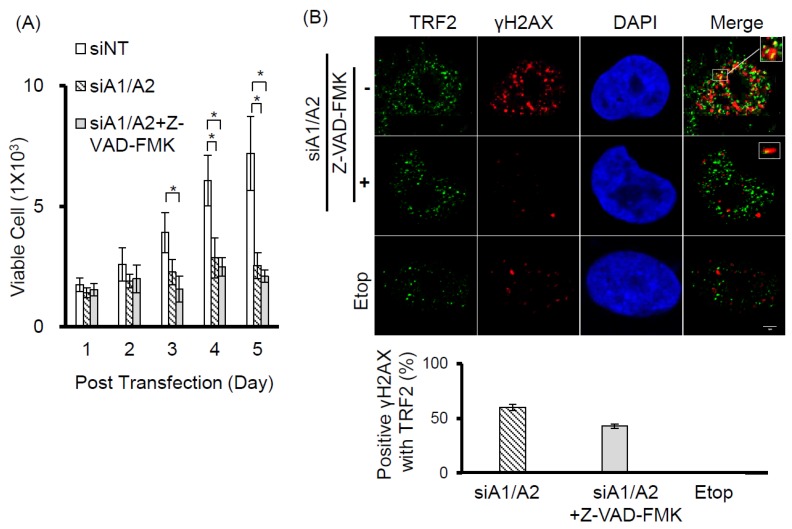
Effects of early apoptosis inhibition on DDR and cell proliferation. (**A**) A549 cells were transfected with siRNA targeting sequences for both hnRNP A1 and A2 (siA1/A2) in the presence or absence of 20 mM of Z-VAD-FMK. Cells transfected with a non-target sequence (siNT) in the absence of Z-VAD-FMK served as a control. The transfected cells were cultured and monitored for cell proliferation using trypan blue staining. The data shown are pooled from 2 independent experiments; (**B**) After 72 h of transfection, the cells were fixed and immunostained for γH2AX (red) and TRF 2 (green), and the nuclei were counterstained with DAPI (blue). The foci of γH2AX that co-localized with TRF2 are indicated by marked squares. Cells treated with 50 μM of etoposide (Etop) for 12 h served as a negative control. The percentage of positive co-localization of γH2AX with TRF2 was determined from analysis of 30 γH2AX-positive nuclei from each experiment. The data shown in the bottom panel are from 2 independent experiments. * *p* < 0.05 versus siNT control. The scale bar equals 2 μm.

Our observation that γH2AX foci were co-localized with telomeres suggests that cells depleted of hnRNP A1/A2 produce dysfunctional telomeres. Because dysfunctional telomeres are known to induce apoptosis [29,33], and because apoptotic DNA fragmentation is known to result in γH2AX phosphorylation [34], this finding suggests that the induction of apoptosis and inhibition of cell proliferation following simultaneous depletion of hnRNP A1/A2 [23,24,32] is caused primarily by dysfunctional telomeres. Alternatively, the formation of dysfunctional telomeres and the induction of apoptosis may also be independent of each other. To address this, we examined the effects of apoptosis inhibition on cell proliferation and the induction of the DDR indicator γH2AX. A549 cells were transfected with siRNAs targeting hnRNP A1 and/or A2 for 48 h then cultured in the presence or absence of the pan-caspase inhibitor Z-VAD-FMK (carbobenzoxy-valyl-alanyl-aspartyl-[O-methyl]- fluoromethylketone) for the indicated times. The transfected cells were then analyzed for activation of apoptosis, expression of γH2AX, and cell proliferation. As shown in Figure 3A, no apoptosis was detected in cells transfected with siNT, siA1 or siA2. In contrast, the activation of caspase-7 and -8, cleavage of poly-(ADP-ribose) polymerase (PARP), and increased levels of γH2AX were detected in cells depleted of hnRNP A1/A2. Moreover, in the presence of Z-VAD-FMK, γH2AX levels were greatly reduced in association with an inhibition of caspase-7 and -8 activation and PARP cleavage, suggesting that γH2AX formation is strongly associated with the induction of apoptosis. However, the inhibition of apoptosis by Z-VAD-FMK did not restore the proliferative capacity of cells depleted of hnRNP A1/A2 (Figure 3B), suggesting that the inhibition of cell proliferation caused by depletion of hnRNP A1/A2 is not primarily attributable to apoptosis. Importantly, although the number of γH2AX foci in hnRNP A1/A2-depleted cells was greatly reduced by an inhibition of apoptosis, the formation of dysfunctional telomeres, as detected by the co-localization of γH2AX with TRF2 (telomeric repeat binding factor 2) in γH2AX-positive nuclei, was unaffected (Figure 3C). To rule out the possibility that the observed failure to restore cell proliferation capacity was attributable to late inhibition of apoptosis, we examined the effects of inhibiting apoptosis at the beginning of siRNA transfection. Similar to the results shown in Figure 3, an early inhibition of apoptosis also failed to alleviate the production of dysfunctional telomeres and inhibit cell proliferation (Figure 4) in hnRNP A1/A2-depleted A549 cells. These results are consistent with the hypothesis that the formation of dysfunctional telomeres is the primary cause of the inhibition of cell proliferation induced by depletion of hnRNP A1/A2. 

### 2.4. Telomerase Dependence of the Induction of DDR and Dysfunctional Telomeres in Cells Depleted of hnRNP A1/A2

**Figure 5:**
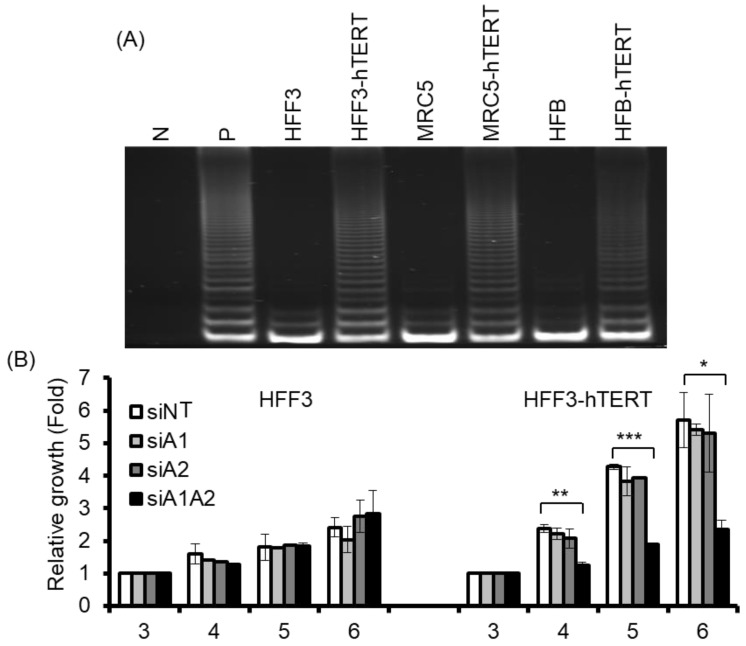

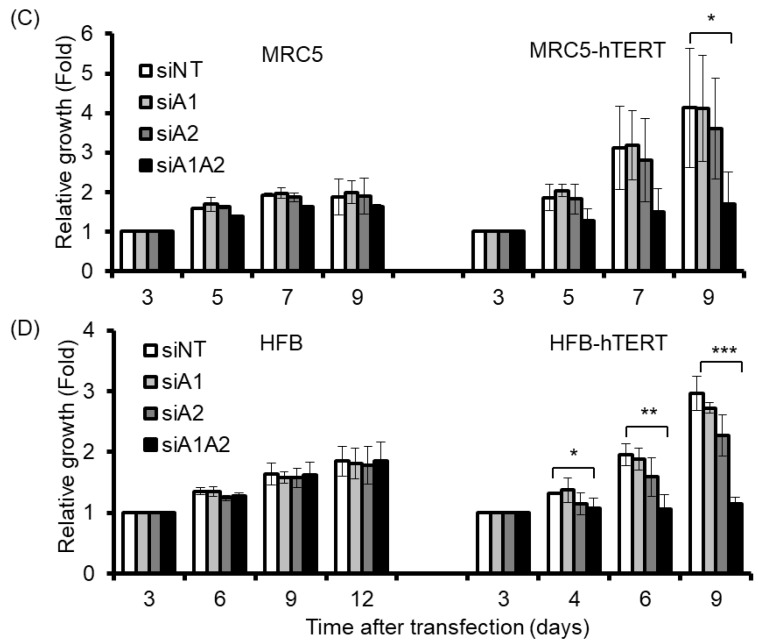
Effects of suppressing hnRNP A1 and/or A2 on cell proliferation of normal fibroblasts and hTERT-immortalized normal fibroblasts. (**A**) Cell lysates from normal fibroblasts (HFF3, MRC5, and HFB) and hTERT-immortalized normal fibroblasts (hFF3-hTERT, MRC5-hTERT, and HFB-hTERT) were assayed for telomerase activity, as described in the materials and methods section. N represents a negative control with no cell extract, while P is a positive control with cell extract from telomerase-positive A549 cells; (**B**–**D**) Normal fibroblasts and hTERT-immortalized normal fibroblasts were transfected with siRNA targeting hnRNPA1 (siA1), hnRNPA2 (siA2), both hnRNP A1 and A2 (siA1/A2), or with a non-targeting sequence (siNT) for 72 h. Transfected cells were cultured in regular medium and monitored for cell proliferation using trypan blue staining. The results shown are pooled from 2 independent experiments. * *p* < 0.05, ** *p* < 0.01 and *** *p* < 0.001 versus siNT control.

**Figure 6:**
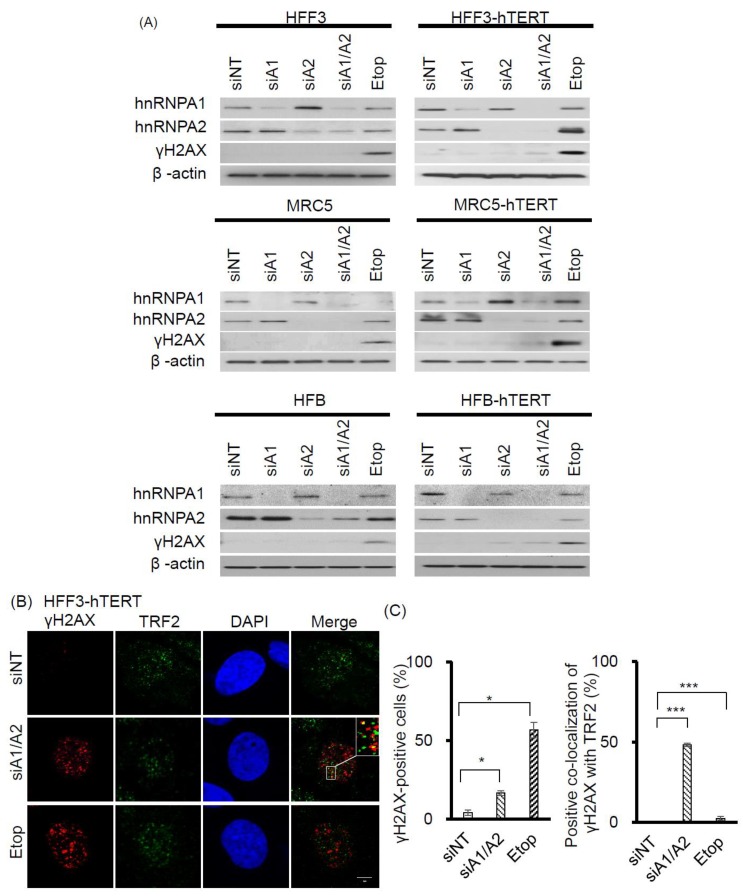
Effects of suppressing hnRNP A1 and/or A2 on DDR in normal fibroblasts and hTERT-immortalized normal fibroblasts. (**A**) Normal fibroblasts (HFF3, MRC5, and HFB) and hTERT-immortalized normal fibroblasts (HFF3-hTERT, MRC5-hTERT, and HFB-hTERT) were transfected with siRNA targeting hnRNPA1 (siA1), hnRNPA2 (siA2), both hnRNP A1 and A2 (siA1/A2), or with a non-targeting sequence (siNT). After 72 h, cell lysates were analyzed for expression levels of hnRNP A1, hnRNP A2, and γH2AX by Western blotting. β-Actin served as a loading control, and cells treated with 50 μM etoposide (Etop) for 12 h served as a positive control; (**B**) Transfected HFF3-hTERT cells were fixed and immunostained for γH2AX (red) and TRF2 (green), and nuclei were counterstained with DAPI (blue). HFF3-hTERT cells treated with 50 μM etoposide (Etop) for 12 h were included as a control; the scale bar equals 5 μm. (**C**) The percentage of nuclei positive for γH2AX staining was determined from analysis of ~100 nuclei from each experiment; the data shown in the left panel are from 2 independent experiments. The percentage of γH2AX co-localized with TRF2 was determined from analysis of 50 γH2AX -positive nuclei from each experiment; the results shown in the right panel are from 2 independent experiments. * *p* < 0.05 and *** *p* < 0.001 versus siNT control.

Although the depletion of hnRNP A1/A2 has been shown to inhibit cell proliferation in most cancer cells, it does not inhibit cell proliferation in normal mortal human cells [24]. One reason for this is thought to be the major differences between normal mortal cells and cancer cells in telomerase activity, which is expressed in most cancer cells, but not in normal somatic cells [35,36]. To test the hypothesis that the expression of telomerase is required for the inhibition of cell proliferation by the depletion of hnRNP A1/A2, we examined the effects of telomerase expression on the sensitivity of normal mortal fibroblasts to the depletion of hnRNP A1 and/or A2. As shown in Figure 5A, while normal fibroblasts HFF3, MRC5, and HFB cells expressed little or no telomerase activity, ectopic expression of hTERT resulted in high expression of telomerase activity comparable to that of cancer cells (A549). Moreover, treatment of these cells with siRNAs targeting hnRNP A1 and/or hnRNP A2 effectively reduced levels of the targeted proteins (Figure 6A). Meanwhile, the depletion of hnRNP A1 or A2 (siA1 or siA2) alone did not inhibit cell proliferation of mortal or hTERT-immortalized normal fibroblasts HFF3, MRC5, or HFB (Figure 5B–D), unlike simultaneous suppression (siA1/A2), which resulted in significant inhibition of cell proliferation in hTERT-immortalized normal fibroblasts, but not mortal fibroblasts (Figure 5B–D). These results confirm earlier findings [24] that simultaneous suppression of hnRNP A1/A2 does not inhibit cell proliferation in mortal normal cells. 

Interestingly, the expression of telomerase activity in normal fibroblasts also rendered these cells sensitive to the inhibition of proliferation following the simultaneous suppression of hnRNP A1/A2 (Figure 5). To determine whether this inhibition of cell proliferation in hTERT-immortalized normal fibroblasts was attributable to the formation of dysfunctional telomeres, we examined the induction and localization of γH2AX in hnRNP A1/A2-depleted cells. As shown in Figure 6A, the depletion of hnRNP A1 or A2 (siA1 or siA2) alone did not induce the formation of γH2AX in normal mortal or hTERT-immortalized fibroblasts HFF3, MRC5, or HFB (Figure 6A), the only exception being very weak induction of γH2AX in the siA2-treated HFB-hTERT cells. Meanwhile, the simultaneous suppression of hnRNP A1 and A2 (siA1/A2) resulted in the induction of low levels of γH2AX in normal hTERT-immortalized fibroblasts, but not in normal mortal fibroblasts. To determine whether this induction of γH2AX was associated with dysfunctional telomeres, we examined the localization of γH2AX in hnRNP A1/A2-depleted HFF3-hTERT cells by immunofluorescence staining. Representative results of γH2AX foci and TRF2 protein co-localization experiments are shown in Figure 6B, with a summary shown in Figure 6C. Although the percentage of γH2AX -positive nuclei in hnRNP A1/A2-depleted hTERT-HFF3 cells (Figure 6C, left panel) was less than that observed in A549 cells (Figure 1C), a similar degree of γH2AX–TRF2 co-localization was observed in γH2AX-positive nuclei from both hnRNP A1/A2-depleted hTERT-HFF3 (Figure 6C, right panel) and A549 cells (Figure 3C). These findings suggest that dysfunctional telomeres are produced in hTERT-HFF3 cells depleted of hnRNP A1/A2.

## 3. Discussion

In this study, we showed that the simultaneous depletion of hnRNP A1 and A2, but not the depletion of hnRNP A1 or A2 alone, induces dysfunctional telomeres and DDR in cancer cells (Figure 1 and Figure 2). The dysfunctional telomeres produced in hnRNP A1/A2 depleted cancer cells were not attributed to short telomeres (Figure 1B), suggesting that there was no dysregulated erosion of telomeres. Because dysfunctional telomeres are known to trigger apoptosis and cell senescence, it is likely that the inhibition of proliferation and induction of apoptosis in cancer cells [23,24,32] following a simultaneous depletion of hnRNP A1/A2 is caused primarily by the formation of dysfunctional telomeres. Consistent with this, we found that the inhibition of apoptosis did not alleviate the production of dysfunctional telomeres (Figure 3C and Figure 4B) or the inhibition of cell proliferation (Figure 3B and Figure 4A).

**Figure 7:**
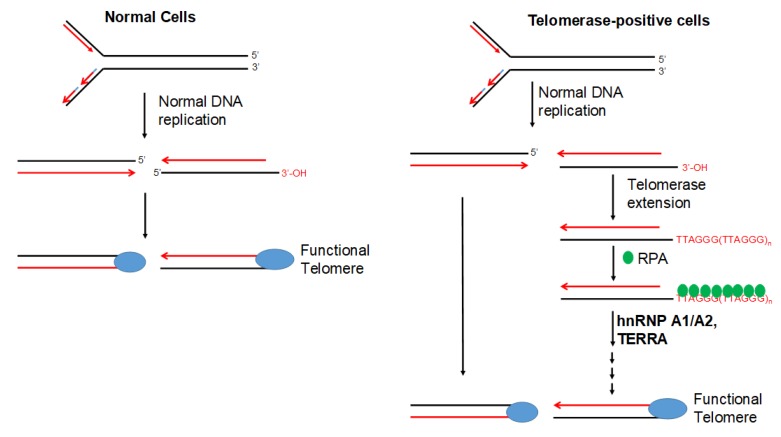
Model of telomerase-dependent maintenance of telomeres. Normal cells do not express telomerase, and the ends of their chromosomes are replicated by regular DNA replication enzymes before being assembled into functional telomeres. In telomerase-positive cells (e.g., cancer cells), the 3′ G-rich single-strand tail can be extended by synthesis of TTAGG repeats, which are thought to be initially bound by RPA. The extended long G-rich strand subsequently serves as a template for lagging-strand DNA synthesis before being assembled into a functional telomere. In this model, hnRNP A1/A2 is thought to facilitate lagging-strand synthesis and/or assembly of a functional telomere structure, possibly by acting together with TERRA to displace RPA.

Our finding that dysfunctional telomeres are produced only in cells depleted of both hnRNP A1 and A2 is consistent with the hypothesis that these 2 proteins perform complementary functions in telomere maintenance. However, the depletion of hnRNP A1/A2 did not inhibit cell proliferation in normal mortal human cells [24]. One reason for this is may be the major difference between normal mortal cells and cancer cells in telomerase activity, which is expressed in most cancer cells, but not in normal mortal cells. We therefore postulate that, in the presence of telomerase activity, the single-strand G-rich 3′ overhang is extended by telomerase, replicated by lagging-strand DNA synthesis, and assembled into functional telomeres (Figure 7). It is likely that hnRNP A1/A2 and TERRA function to displace RPA [30], facilitating a lagging-strand DNA synthesis and/or the assembly of functional telomeres. Consistent with this, we revealed that, while a simultaneous depletion of hnRNP A1/A2 did not induce DDR or inhibit cell proliferation in mortal HFF3, MRC5, or HFB cells, an immortalization of these cells by ectopic expression of hTERT rendered these telomerase-expressing normal cells sensitive to the inhibitory effects of simultaneous depletion (Figure 5). In this context, it is worthy to note that although dysfunctional telomeres are produced in the hTERT-immortalized HFF3 by simultaneous depletion of hnRNP A1/A2 (Figure 6), no apoptosis induction was observed in these depleted cells (unpublished results). It is likely that the dysfunctional telomeres produced in hTERT-immortalized normal cells only induce cell senescence. Lastly, it should be noted that some cancer cells employ alternative lengthening of telomeres (ALT) during telomere length maintenance. ALT is a homology-directed recombination-dependent replication pathway that utilizes telomeric templates for synthesis [37]; however, its precise protein requirements remain unknown. Whether or not hnRNP A1/A2 are essential components of the ALT therefore requires further analysis.

This study has substantiated for a role of hnRNP A/Bs in telomere maintenance. While the ability of hnRNP A/Bs to bind to the single-stranded telomeric repeat sequence of DNA and TERRA may account for the major function of these proteins in telomere maintenance, little is known if these proteins may also be involved in the regulation of hTERT expression or the recruitment of telomerase to telomeres. The hnRNP A1 has been reported to interact with the human telomerase holoenzyme and stimulate telomerase activity [15,18]. A splice variant of hnRNP A2, hnRNP A2*, has been shown to bind telomeric DNA and telomerase in vitro and to unfold telomeric G-quadruplex DNA to exposes 5 nt of the 3’ telomere tail [38]. Therefore, both hnRNP A1 and hnRNP A2 appear to participate at multiple steps of telomere maintenance.

The specific expression of telomerase in most cancers suggests the potential application of telomerase targeting in cancer therapy. Various telomerase-based therapeutic strategies have been explored in the past, such as immunotherapy, hTERT-promoter–based gene therapy, and the inhibition of telomerase activity [39,40,41]. Our demonstration of a role for hnRNP A1/A2 in telomerase-dependent telomere maintenance therefore suggests a novel approach for telomerase-based cancer therapy. The innhibition of telomerase-dependent telomere maintenance (Figure 7) is anticipated to result from the formation of dysfunctional telomeres, which can rapidly inhibit cell proliferation and induce apoptosis only in telomerase-positive cancer cells. A detailed molecular understanding of telomerase-dependent telomere maintenance as well as an analysis of the agents that specifically interfere with this process should therefore be examined in the future.

## 4. Materials and Methods 

### 4.1. Culture Media, Antibodies, and Oligonucleotides 

The culture media and fetal bovine serum were purchased from Life Technologies (Grand Island, NY, USA). The antibodies against the following proteins were obtained from the indicated vendors: cleaved PARP (Asp214) and γH2AX (9718), (Cell Signaling Technology, Temecula, CA, USA); hnRNP A1 (F-8) and hnRNP A2 (EF-67) (Santa Cruz Biotechnology, Inc., Santa Cruz, CA, USA); TRF2 (Millipore, Billerica, MA, USA); β-actin (Sigma-Aldrich, St. Louis, MO, USA); MDC-1 (M2444) (Sigma-Aldrich); caspase-7 (9492), -8 (9746), and PARP (9542) (Cell Signaling Technology). The FITC 488–OO-(CCCTAA)_3_ PNA probe (F1009) was obtained from Panagene (Daejeon, Korea), and etoposide (E1383) was purchased from Sigma-Aldrich. Superscript III reverse transcriptase, TRIzol reagent, and antibiotics were obtained from Gibco-BRL (San Francisco, CA, USA). The broad-spectrum caspase inhibitor Z-VAD-FMK was purchased from Promega (Fitchburg, WI, USA). Gel electrophoresis reagents were obtained from Bio-Rad (Berkeley, CA, USA). 

### 4.2. Cell Lines and Culture 

The NSCLC cell line, A549, was obtained from the American Type Culture Collection (Manassas, VA, USA). The CL1-5 cell line was derived from NSCLC CL1-0 cells by the selection of increased invasion ability using a Transwell plates [42]. CL1-5 and A549 cells express wild-type EGFR [43]. Normal human lung fibroblasts (MRC5) were purchased from The Food Industry Research and Development Institute in Taiwan. Human skin fibroblasts (HFB) were kindly provided by Dr. P. C. Yang of Taiwan University. The immortalization of MRC5 and HFB cells by ectopic expression of hTERT (human telomerase reverse transcriptase) was carried out as described previously for normal human foreskin fibroblasts (HFF3) [44]. Normal human fibroblasts (HFF3, MRC5, HFB) and hTERT-immortalized normal fibroblasts (HFF3-hTERT, MRC5-hTERT, and HFB-hTERT) were cultured in Dulbecco’s modified Eagle’s medium supplemented with 10% fetal bovine serum, 100 units/mL penicillin, 100 units/mL streptomycin, and 0.25 mg/mL amphotericin. Culture of the human lung cancer cell lines A549 and CL1-5, and human tongue squamous carcinoma cell line SAS was carried out as described previously [32,45]. Cells were grown at 37 °C in a humidified 5% CO_2_ incubator.

### 4.3. RNA Interference 

Target genes were downregulated by RNAi-mediated inhibition of mRNA expression using a mixture of 4 small interfering RNAs (siRNAs) for each target gene (ON-TARGETplus SMARTpool; Dharmacon, Lafayette, CO, USA) [32]. The siGENOME nontargeting siRNA pool (Dharmacon) was used as a control. The siRNA sequences were run through BLAST searches to ensure that each siRNA targeted only 1 human gene. The 4 siRNAs targeting human hnRNP A1 mRNA (GenBank accession No. NM_002136) covered the following sequences: A1-1, CGGAAACCUUGGUGUAGUU (nucleotides 1545–1563); A1-2, GGGAAUGAAGCUUGUGUAU (nucleotides 1709–1727); A1-3, CAACUUCGGUCGUGGAGGA (nucleotides 746–764); and A1-4, UAGAAUUCCUUCAGGGUGA (nucleotides 1468–1486). The 4 siRNAs targeting hnRNP A2 mRNA (GenBank accession no. NM_002137) covered the following sequences: A2-1, CGGUGGAAAUUUCGGACCA (nucleotides 825–843); A2-2, GCUGUUUGUUGGCGGAAUU (nucleotides 519–537); A2-3, GGAGAGUAGUUGAGCCAAA (nucleotides 443–461); and A2-4, GAGGAGGAUCUGAUGGAUA (nucleotides 863–881). Transfection was performed using Dharmafect 1 transfection reagent (Dharmacon) according to the manufacturer’s instructions. Briefly, exponentially growing cells were seeded in regular growth medium without antibiotics at 40–50% confluence. After 24 h, cells were transfected with siRNAs and incubated for indicated times.

### 4.4. Cell Proliferation Assays 

Cell proliferation was assayed using an MTT assay kit (Sigma-Aldrich) and by staining with trypan blue (to determine the number of viable cells) as described previously [32].

#### Western Blotting 

Western blotting was performed as described previously [32].

### 4.5. Telomerase Assay

A PCR-based telomeric amplification protocol [35] was used to assay telomerase activity. The preparation of cell extracts, PCR amplification conditions, and analysis of PCR products by electrophoresis on polyacrylamide gel were performed as described previously [46].

### 4.6. Determination of Telomere Length 

Measurements of telomeric restriction fragment (TRF) length were performed using a TeloTAGGG Telomere Length Assay Kit (Roche, Basel, Switzerland) as described previously [20]. Quantification of telomere length was performed using ImageQuant software (GE Healthcare Life Sciences, Molecular Dynamics GmbH, Krefeld, Germany) and TELORUN.

### 4.7. Immunofluorescence Confocal Microscopy 

Immunofluorescence staining of target proteins was performed as described previously [17] with some modifications. Briefly, cells on slides were fixed in 4% paraformaldehyde, permeabilized, and stained with anti-γH2AX, anti-TRF2, or anti-MDC1 antibodies. After washing three times with phosphate buffer saline (PBS) containing 0.1% Triton X-100, cells were incubated with Texas red- or fluorescein isothiocyanate (FITC)-conjugated secondary antibodies (Molecular Probes, Eugene, OR, USA) for 45 min at room temperature. Staining of interphase nuclei of telomeres by fluorescence in situ hybridization (FISH) was performed as described previously [47] with some modifications. Briefly, dehydrated slides were overlaid with 0.8 μg/mL of FITC 488–OO-(CCCTAA)_3_ PNA probe (Panagene) in the PNA hybridization solution (10 mM NaHPO_4_, 10 mM NaCl, 20 mM Tris, pH 7.5, 70% [*v*/*v*] formamide), incubated at 80 °C for 5 min, and hybridized at room temperature for 16 h. Slides were washed with PNA wash I (PBS/0.1% Tween-20), followed by PNA wash II (2X SSC/0.1% Tween-20), and mounted with Fluoroshield containing 4′,6-diamidino-2-phenylindole (DAPI; F6057, Sigma-Aldrich). Immunofluorescence analyses were carried out under a confocal microscope (LSM 700; Carl Zeiss, Jena, Germany) and the results were processed using Zen 2009 software (Carl Zeiss).

### 4.8. Statistics 

All data are presented as mean ± SD. Statistical comparison of multiple-groups results was performed by One-Way ANOVA (Analysis of Variance), followed by *post hoc* Mann–Whitney *U* test. A *p*-value of < 0.05 was considered statistically significant. 

## 5. Conclusions

This study reported for the first time that hnRNP A1 and A2 function in the maintenance of telomeres in telomerase-expressing cells, explaining earlier findings that simultaneous suppression of hnRNA1 and A2 inhibits cell proliferation and induces apoptosis in cancer cells, but not in normal mortal cells. Because the formation of dysfunctional telomeres rapidly inhibits cell proliferation and induces apoptosis, and because telomerase is expressed in most cancer cells but not in normal cells, inhibition of telomerase-dependent maintenance of telomeres could provide an attractive novel approach for telomerase-based cancer therapy.
